# “3D, human renal proximal tubule (RPTEC-TERT1) organoids ‘tubuloids’ for translatable evaluation of nephrotoxins in high-throughput”

**DOI:** 10.1371/journal.pone.0277937

**Published:** 2022-11-21

**Authors:** Sarah E. Vidal Yucha, Doug Quackenbush, Tiffany Chu, Frederick Lo, Jeffrey J. Sutherland, Guray Kuzu, Christopher Roberts, Fabio Luna, S. Whitney Barnes, John Walker, Pia Kuss

**Affiliations:** 1 Novartis Institutes for BioMedical Research-San Diego, La Jolla, CA, United States of America; 2 Novartis Institutes for BioMedical Research-Cambridge, Cambridge, MA, United States of America; Universidade Federal do Rio de Janeiro, BRAZIL

## Abstract

The importance of human cell-based *in vitro* tools to drug development that are robust, accurate, and predictive cannot be understated. There has been significant effort in recent years to develop such platforms, with increased interest in 3D models that can recapitulate key aspects of biology that 2D models might not be able to deliver. We describe the development of a 3D human cell-based in vitro assay for the investigation of nephrotoxicity, using RPTEC-TERT1 cells. These RPTEC-TERT1 proximal tubule organoids ‘tubuloids’ demonstrate marked differences in physiologically relevant morphology compared to 2D monolayer cells, increased sensitivity to nephrotoxins observable via secreted protein, and with a higher degree of similarity to native human kidney tissue. Finally, tubuloids incubated with nephrotoxins demonstrate altered Na+/K+-ATPase signal intensity, a potential avenue for a high-throughput, translatable nephrotoxicity assay.

## Introduction

The average time it takes for a new drug to be developed is 14 years. The average cost is over $ 2.6 billion USD. For every 10k drugs investigated in early stages, only 1 makes it through the clinic [1]. Once in clinical trial phases II and III, an estimated 95% of candidates are eliminated—with an estimated 30–40% of failures in clinical trial phases II and III are due to toxicity [2,3], with some estimates as high as 60% for phase II trials [4]. Estimated 21% of drug withdrawals are nephrotoxic/hepatic issues [5].

One reason for the failure of drugs to make it to market is issues with toxicity and/or off-target toxicity; this is due to many intersecting issues, including relevance of animal models to human biology, lack of a reliable human *in vitro* models, lack of robust biomarkers which translate from *in vivo* studies, and possible patient-specific genetic differences which impact tolerability and responsiveness to treatments [6]. Late-stage failed compounds typically underestimate renal toxicity potential [7]. Further, renal toxicity acts as a dose-limiting parameter for many treatments [8,9].

Considering the wealth of data accumulating suggesting that our preclinical efforts into toxicity are not robust enough to predict toxicity in humans, it is likely we need to rethink our approach as an industry to develop a predictive *in vitro* human assay [2,3,5,10]. Of all drugs available on the market, ~33% are renally cleared and ~25% are excreted in the urine unchanged [11]. Proximal tubule epithelial cells (PTECs) are particularly susceptible to damage because secreting drugs is an active process, requiring many drug transporters to function together to eliminate drugs and their metabolites.

PTECs tend to accumulate toxins and serve as the kidney’s primary method to eliminate (secrete) toxins [12]. After plasma is filtered in the glomerulus, then enters the Bowman’s capsule the remaining 80% flows through peritubular capillaries, where the proximal tubule performs its key function of clearance via activation of basolateral transporters [12]. Secretion at the proximal tubule is the main mechanism of elimination of toxins, and hence why the site is particularly vulnerable to drug-induced toxicity [6,12]. Abnormalities in renal transporters can lead to drug-induced nephrotoxicity [11].

There is a pressing need to develop an accurate, human, *in vitro*-based model that can predict human toxicity response and intervene at the earliest stages of drug development. In 2D, human primary renal cells quickly lose their differentiation and functionality; common alternatives such as MDCK, CHO, HEK293 although frequently used, lack transporter function and specific biomarkers, therefore have less-than-ideal physiological relevance. Significant effort to develop technologies which better approximate the physiology of human kidneys have been made, through advancements such as iPSC-derived organoids or complex organ-on-chips [13–17]. Additionally, many groups have explicitly considered differences between 2D and 3D culture methods to emphasize the likely importance of 3D culture for relevant systems [18–20]. Although these studies have helped to identify crucial aspects of biology that are likely required for a relevant *in vitro* model, no 3D system is currently feasible for high-throughput assays making them unrealistic for drug discovery efforts [21].

RPTEC/TERT1 cells are RPTEC cells that have been immortalized by pLXSN-human telomerase (hTERT) retroviral transfection while still maintaining proximal tubule phenotype and being able to be expanded indefinitely. They have been researched as a possible model system for many renal applications due to their stability and the fact that they are primary, human cells [22]. A major aim of this study is to validate whether these cells can be utilized for forming tubuloids, and whether they will express key biomarkers with crucial renal function (examples: apical brush border, transporters, cell polarization, differential response to compounds). Interestingly, prior work has demonstrated that these cells can spontaneously organize into tubules [20] as well as seeming to have the ability to form spheroids when cultured in 3D [23]. Several groups have already investigated these cells for their potential utility for identifying toxicity [24–26].

The RPTEC-TERT1 tubuloids demonstrated several advantages over 2D (monolayer) RPTEC-TERT1; most importantly, that the 3D tubuloids were more similar genetically to human kidneys, with nephrotoxicity results that also aligned with *in vivo* animal studies. Further, the tubuloids are polarized, with a clearly defined lumen, and stain for several key transporters (including Na+/K+-ATPase, SGLT2, OAT1, OCT2, Aquaporin-1, Aquaporin-3), with clear primary cilium growing on the interior (apical) side of the lumen, and the tubuloids also stain for multiple markers which have been proposed as key markers to identify kidney injury (KIM-1, Osteopontin, HMOX)—emphasizing the utility of this system as a tool in drug development [7,27].

The objective of the study is to establish kidney tubuloids (‘proximal tubule organoids’) from primary human kidney cells (RPTEC/TERT1), to be adapted for high-throughput applications. Prior work has identified predictive compound sets amenable to nephrotoxicity screening, containing both toxic compounds as well as a variety of non-toxic controls [27,28]. To date there exist no current treatments for end-stage kidney disease. The opportunity to reliably predict nephrotoxicity, and thereby methods to prevent/inhibit or reverse/regenerate damage could be a paradigm shift to the field of drug discovery.

## Methods

### Materials

Key materials are described in S1 Table and other supporting information.

### Cell culture

RPTEC-TERT1 (ATCC) were cultured according to all manufacturer recommendations, in a 37°C incubator with 5% CO_2_.

#### To prepare 3D cultures

To keep passage numbers the same throughout experiments (passage 9), RPTEC-TERT1 frozen vials were seeded directly into culture. First, cells were counted (with viability confirmed to be above 80%) and reconstituted to 6.5 *10^5^ cells/mL. Matrigel (growth factor reduced, Corning) was kept on ice during preparation. Reconstituted cell solution was mixed with matrigel at 50% (v/v), mixed well, and then plated into CellCarrier Ultra 384-well plates (Perkin Elmer) at 15 μL/well. The plates were tapped to ensure even coating of the cell-matrigel solution, then allowed to cure in a 37°C, 5% CO_2_ incubator for at least 30 minutes. Once gelation occurred, 75 μL of RPTEC-TERT1 media was plated into each well.

#### To prepare 2D cultures

Using the same vial as the 3D cultures, cells were plated using 15 μL/well of cells at 6.5 *10^5^ cells/mL. Then, 75 μL of RPTEC-TERT1 media was plated into each well.

#### Injury assay

After 7 days in culture, media is exchanged using an automatic liquid handler. On day 10 of culture, media is aspirated and replaced with either control, or compound-containing media at a predefined dose (Table 1) and incubated for 4, 24, or 48 hours.

**Table 1:** Media conditions for injury assay.

Media condition for dosing	Concentration (μM)
Standard RPTEC-TERT1 media control	N/A
Cadmium chloride	4.5, 9, 55 (low, mid, high)
Cisplatin	10, 30, 50 (low, mid, high)

### Genetic analysis

Samples (2D and 3D) were grown in 24-well culture to ensure RNA-yield for genetic analysis. Samples were isolated using Trizol at 500 μL/well (24-well plate), with gentle homogenization. The resultant lysate was then frozen at -80°C until RNA extraction.

#### RNA extraction

Samples were homogenized using QiaShredder according to manufacturer protocol (Qiagen); RNA was immediately extracted using DirectZol Prep (Zymo) according to manufacturer protocol and eluted in water.

#### RNASeq (bulk)

One hundred to two hundred nanograms of total RNA was used to make libraries using Lexogen Quantseq reagents. Libraries were sequenced using 50 bp single reads on an Illumina HiSeq 1000 to an average depth of 15 million reads (mapped to genes) per sample. For all samples, bcl2fastq2-2.20.0.422–2 was used to make fastqs. Reads were aligned with STAR v 2.6.1d against the version 101 genome and gtf from Ensembl [29]. RSEM version 1.2.28 was used to assigned reads to genes [30]. DESeq2 version 1.28.0 was run in R 4.0.2 to calculate differentially expressed genes [31]. Gene changes were calculated via “DESeq2” package in R. Orthologs were identified via “biomaRt” package in R and common genes were considered in the gene expression profile comparisons.

### Phenotypic, high content imaging

#### Antibody staining

For 384 well culture, media was aspirated then plates were fixed in 4% (v/v) paraformaldehyde (PFA) solution (in phosphate buffered saline (PBS)) with gentle shaking. Samples were then washed three times with PBS using an automated plate washer; following the final wash samples were incubated with blocking solution (4% bovine serum albumin (BSA) (w/v), 0.5% Triton (v/v) in PBS) for at least overnight at 4°C (35 μL/well).

Blocking solution was aspirated the following day, and then primary antibodies were added (35 μL/well), prepared in antibody buffer (2% BSA, 0.25% Triton in PBS) and incubated with gentle shaking throughout the day and moved to 4°C storage overnight.

Primary antibodies were then aspirated, and plates were washed with PBS– 0.05% (v/v) Tween 20 (PBST) solution three times. Secondary antibodies, prepared in antibody buffer, were added (35 μL/well) and incubated with gentle shaking throughout the day and moved to 4°C storage overnight. Antibodies/dyes described in S2 Table. On the final day, plates were washed six times with PBST, and then sealed.

#### Tubuloid image intensity

Images were first corrected using the BaSiC background correction method [32]. Tubuloids were then segmented using cellpose with an approximate diameter of 100 pixels, and Na+/K+-ATPase signal intensity was measured within each detected tubuloid [33]. The primary readout for this assay, 95th percentile Na+/K+-ATPase Tubuloid intensity, was calculated for each well by pooling the pixel intensity values of every segmented tubuloid, then taking the 95th percentile of this pool.

### Protein assays

A custom Luminex assay (Biotechne) was prepared for initial studies comparing 2D and 3D systems, the assay was prepared for 96-well culture testing supernatant for 15 injury biomarkers (S3 Table). Assays were performed according to manufacturer specifications and read using a FlexMap 3D (RnD Systems).

Samples were normalized via total protein content which was determined by Pierce Rapid Gold BCA protein assay kit (ThermoFisher). The normalized value was used for further analysis.

## Results

### Phenotypic: Development of 3D human proximal tubule organoids ‘tubuloids’ for high throughput screening

**Fig 1:**
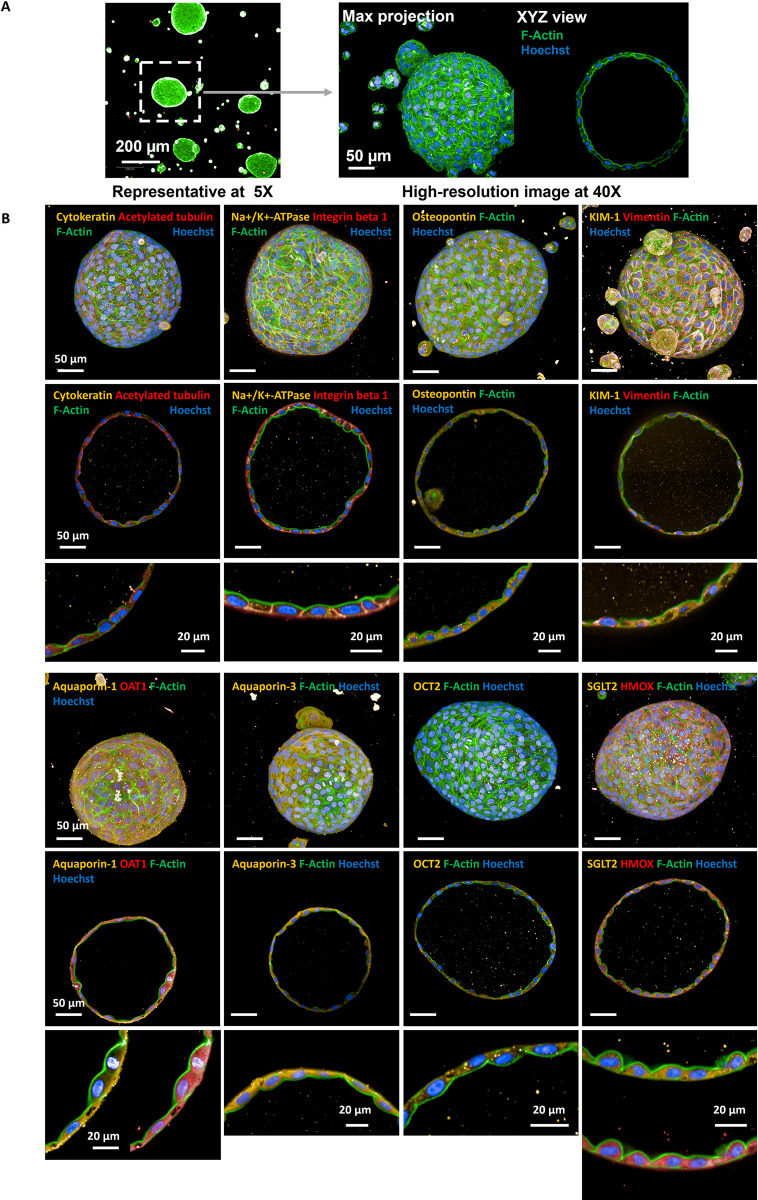
High throughput, high content imaging of tubuloids. (A) Tubuloids cultured in 384-well plates were imaged via Preciscan algorithm (Opera Phenix, Perkin Elmer), wherein individual tubuloids of predetermined diameter, morphology, and quantity per-well were identified and captured in high resolution. (B) Tubuloids display several key biomarkers for proximal tubules including KIM-1, Aquaporin-1, Aquaporin-3, OCT2, OAT1, SGLT2, Na+/K+-ATPase, Integrin beta 1, Acetylated tubulin, Vimentin, Cytokeratin, Osteopontin and HMOX1, and exhibit hollow lumens. Polarity is evident for several markers, including primary cilia which face the interior lumen, as well as Na+/K+-ATPase and integrin beta 1 on the basolateral side.

When cultured in 50% (v/v) growth-factor reduced matrigel, RPTEC-TERT1 cells self-organize into large diameter tubuloids with lumens over the course of 10 days (Fig 1). The tubuloids are cultivated on 384-well plates, making the system highly amenable to automation and high-throughput screening platforms. Tubuloids were stained with several key proximal tubule biomarkers and visualized via high-content imaging (Opera Phenix, Perkin Elmer) to distinguish key features. Importantly, the tubuloids are polarized, exhibit important proximal tubule markers, and display a lumen, enabling physiologically relevant flow/transport from the basolateral to apical side. The biomarkers selected and their membrane location are specified in Table 2, with control staining in 2D cells explored in S1 Fig.

**Table 2:** Tubuloid biomarkers and respective membrane location.

Biomarker	Membrane location
F-Actin	Intracellular
Cytokeratin	Intracellular
Acetylated tubulin	Intracellular
Na+/K+-ATPase	Basolateral
Osteopontin	Intracellular
KIM-1	Intracellular or membrane
Vimentin	Intracellular/intermediate filaments
Aquaporin-1	Basolateral and apical
OCT-1	Basolateral
Aquaporin-3	Basolateral
OCT-2	Basolateral
SGLT2	Apical
HMOX	Intracellular or membrane

### Differences between 2D and 3D morphology

**Fig 2:**
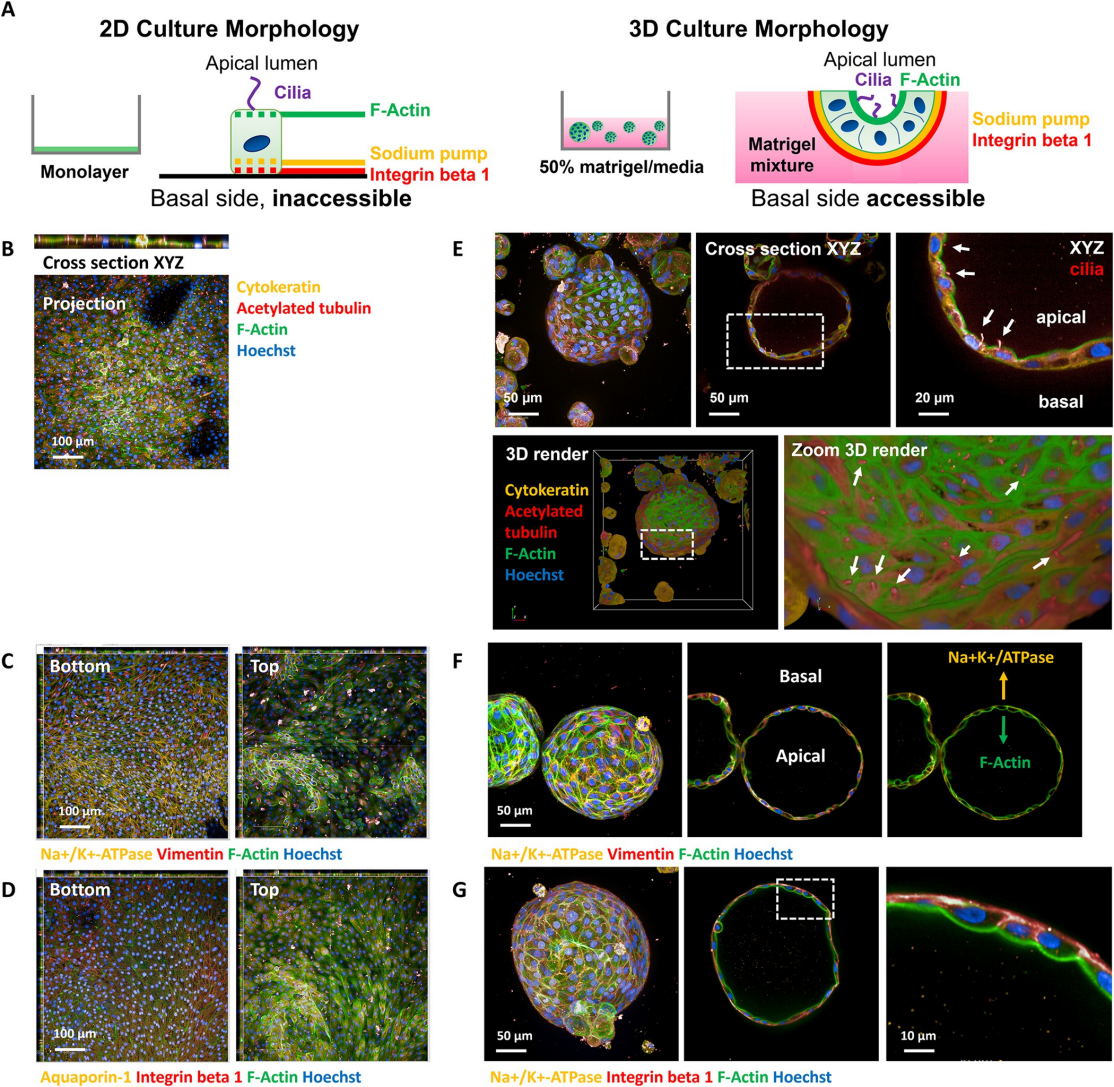
Differences between 2D and 3D culture method. (A, D) In 2D, cilia are present, pointing upwards into the well and are susceptible directly to toxin addition whereas in 3D, the cilia are facing the apical lumen of the tubuloid (white arrows). (B, E) A key transporter (Na+/K+-ATPase) is basolaterally expressed, whereas in 2D while also basal is inaccessible against tissue-culture plastic. (C, F) Similarly, integrin-beta 1 is basolaterally expressed in 3D and 2D, however the 2D basal side is inaccessible whereas in 3D it is accessible.

A key advantage of the tubuloid system for high-throughput screening is that the morphology is effectively opposite of 2D monolayer systems, *i*.*e*., that when compound is applied to the 2D system it is in contact with the apical side of the cells, whereas in the 3D tubuloid system it is in contact with the basolateral side—not the apical side (Fig 2A). This distinguishing characteristic of the assay is evident, with primary cilia visible on the apical lumen in 3D, but in 2D while the cells are polarized, the cilia are directly accessible (Figs 2B and S2). The basolateral side of the 3D tubuloid is on the exterior surface (Fig 2C and 2D), whereas in the 2D monolayer the basolateral side is against the tissue-culture plastic (inaccessible).

### Injury design

To determine which condition would be the most predictive for a nephrotoxicity readout, treatment with known nephrotoxins cisplatin and cadmium chloride were compared with respect to sample type (2D or 3D), time, and dose (Table 3). RNASeq was performed on bulk samples (2D, 3D) on low dose nephrotoxins (cisplatin, cadmium chloride) with respect to time; the secreted protein analysis (2D, 3D) was performed on low, mid, and high dose of the nephrotoxins (cisplatin or cadmium chloride).

**Table 3:** Sample table and description of data collected.

Sample	Time-point	Condition	Data collected
Extracted RNA (2D, 3D)	4 h, 24 h, 48 h	Control (media) Nephrotoxin treatment (low dose of cisplatin (10 μM) or cadmium chloride (4.5 μM))	Bulk RNASeq
Supernatant (2D, 3D)	4 h, 24 h, 48 h = 15-plex, 96-well assay (marker selection) 4 h = 5-plex, 384-well assay (nephrotoxicity screen)	Control (media) Nephrotoxin treatment (low, mid, and high dose of cisplatin (10, 30, 50 μM) or cadmium chloride (4.5, 9, 55 μM))	Luminex assay (protein concentration)
Images (3D)	24 h	Control (media) Cisplatin Cadmium chloride	High content imaging analysis (Na+/K+-ATPase intensity)

### Protein changes upon injury

**Fig 3:**
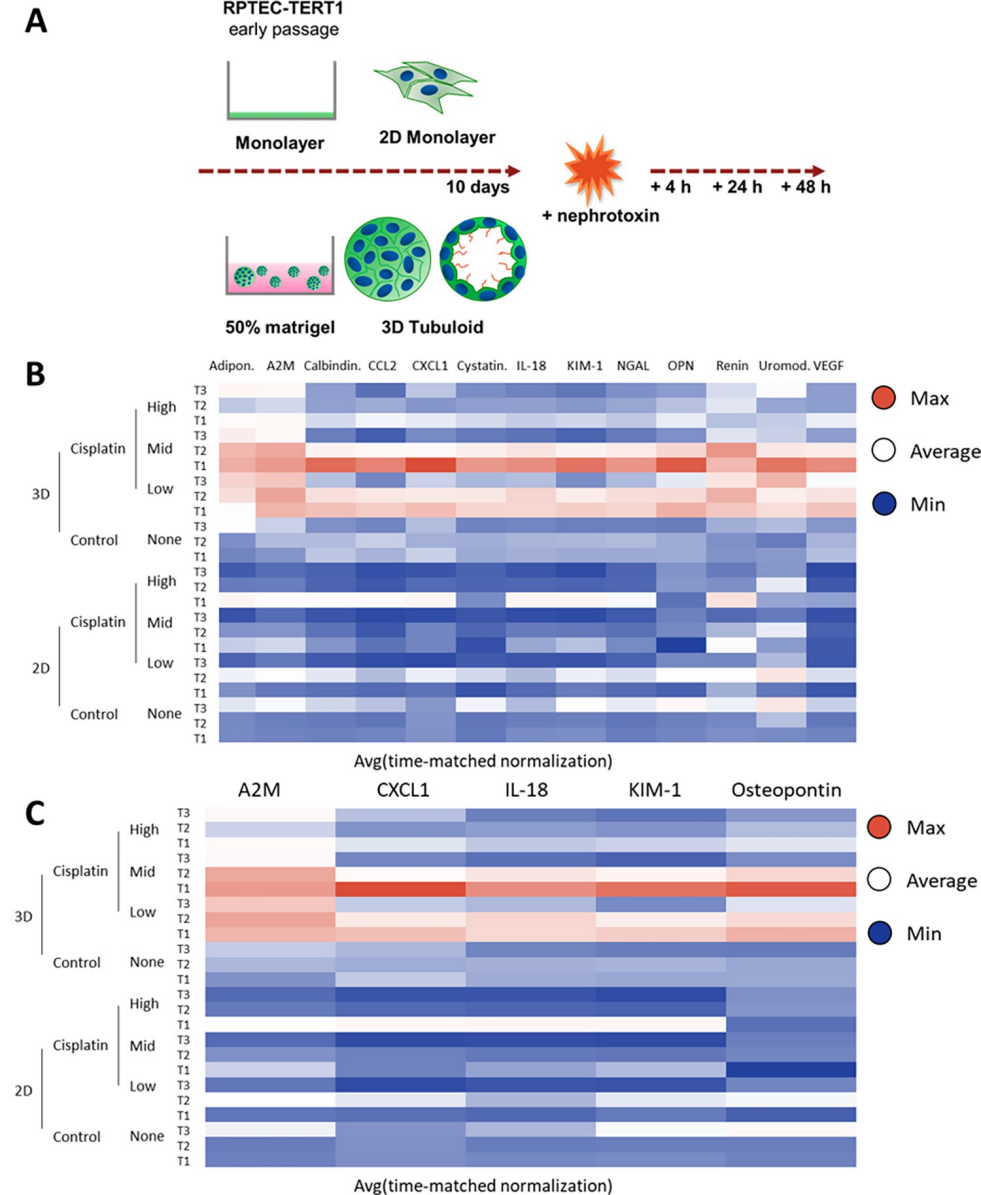
96-well Luminex assay on cisplatin injured samples in 2D and 3D. (A) Injury assay design in 2D and 3D compares cells in monolayer to tubuloids following incubation with cisplatin at pre-determined timepoints. (B) All targets with signal from a 15-plex assay (2 targets, beta-2-microglobulin and TFF3, were not detected) with respect to 2D or 3D samples, and concentration of cisplatin, over time emphasized that 3D tubuloids had higher detectable proteins indicating injury from the 3D tubuloids than the 2D monolayer (C) The highest signal biomarkers were A2M, CXCL1, IL-18, KIM-1, and Osteopontin.

The 2D and 3D system were directly compared for the ability to detect secreted proteins indicating kidney injury. Following incubation with cisplatin, a well-described nephrotoxin, the supernatant was collected, and concentration was determined via Luminex (Fig 3A). In general, it was evident that overall signal for proteins indicating injury was higher and more evident in 3D than in 2D. Further, low and mid dose had the highest signal across all markers in 3D, whereas the signal appeared to be ablated in the high dose most likely due to the toxic effect of the treatment (Fig 3B); the injury proteins with the highest signal in 3D were A2M, CXCL1, IL-18, KIM-1, and Osteopontin (Fig 3C).

### Genetic changes upon injury

**Fig 4:**
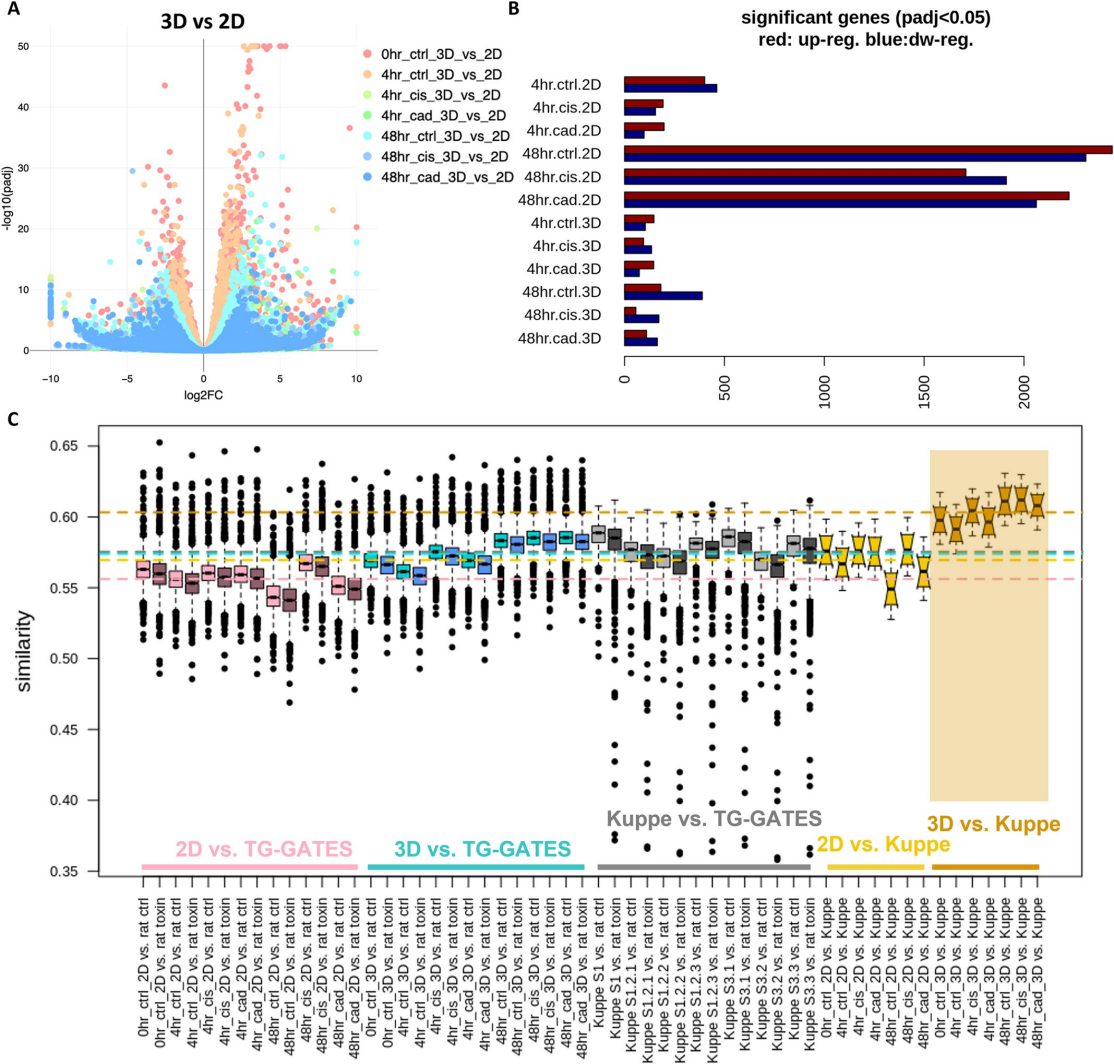
3D tubuloids demonstrate higher similarity to human kidney tissue than 2D monolayer via RNASeq. (A) Visualization of the RNASeq dataset separated by 3D vs 2D for all samples: Gene changes in 3D model against 2D model are shown; |log2FC| higher than 10 is set to 10 and -log10(padj) higher than 50 is set to 50. (B) Number of differentially expressed genes (padj < 0.05) against the base line (0hr) are shown; red bar: Up-regulated genes, blue bar: Down-regulated genes. (C) Comparison of the expression profiles: Expression profiles of 2D and 3D models, proximal tubules subtypes from human kidney scRNAseq (Kuppe et al.; subtypes: S1, S1.2.1, S1.2.2, S1.2.3, S3.1, S3.2, S3.3), and rats from the TG-GATES data set are compared. Compound treated and un-treated profiles are separated in the comparison and similarity score is the Spearman correlation of expression levels. Box plots are showing distribution of comparison of one profile from one data set against one profile from the other data set; notches indicate 95% confidence interval; the average of each dataset is represented by a dashed, colored line. Rat genes are converted to human genes and common genes in two sets are compared in the analysis. The highest similarity of any dataset was the 3D RNASeq to Kuppe. This indicates that the 3D dataset may be more closely relevant to human kidney tissue than 2D.

RNAseq analysis was performed on 3D tubuloids and 2D monolayer cells following incubation with media, cadmium and cisplatin at 0, 4 and 48 hours to compare gene expression signatures to known nephrotoxicology data. Under each treatment condition, changes in gene expression were determined by comparing expression vs. untreated cells at baseline (0 hours). For all 3 treatment conditions (media, cadmium, or cisplatin), a large proportion of genes were significantly changed (p-adj < 0.05) at 48 hours under 2D conditions, but not under 3D conditions (Fig 4B). This suggested that cells cultured under 3D conditions remained more stable and similar to their baseline state in the 3D model and were less susceptible to perturbation. Further, when compared to available nephrotoxicology data, the 3D model demonstrated the highest degree of similarity to data from human renal tissue, with the lowest similarity being the 2D model compared to TG-GATES (comprised of rat *in vivo* data) (Fig 4C) [34].

We hypothesized that compound-induced perturbation of gene expression in our 3D system would better represent changes observed for the same compound *in vivo*. In the absence of human kidney gene expression datasets pre- and post- drug treatment, we leveraged a large compendium of rat kidney gene expression data from the open TG-GATEs repository, available via the TXG-MAPr web application (https://txg-mapr.eu/WGCNA_kidney/TGGATEs_KIDNEY, accessed 7/14/2021) [35]. This database contains kidney gene expression results for 41 drugs and other toxicants, administered to rats at 3 doses for durations ranging from 3 hours to 29 days, for a total of 975 compound-dose-time treatment conditions compared to time-matched vehicle controls. Human gene expression from our culture model were mapped to rat genes using the RGD database and uploaded to the TXG-MAPr application [36].

TXG-MAPr converts log fold change for individual genes into kidney co-expression scores summarizing the treatment-induced changes in expression across 347 networks. The profiles for cadmium and cisplatin after 48 hours in culture were compared to 975 rat kidney profiles. The 10 most similar profiles for cisplatin-3D included cisplatin at various times / doses (7 hits), carboplatin (1 hit) and puromycin aminonucleoside (1 hit) (Table 4). All these treatments cause renal tubule degeneration and/or necrosis when administered to rats according histopathology findings from TG-GATES. The second most similar hit was cadmium under 3D conditions, underscoring the utility of gene expression similarity for grouping treatments causing similar toxicities. In contrast, cisplatin-2D retrieved a diverse set of rat kidney profiles with lower similarities, including 17-methyltestosterone, cyclophosphamide, hexachlorobenzene among others; none of these treatments caused tubule degeneration *in vivo* at the administered dosages. The most similar rat kidney profiles vs. cadmium-3D included various compounds. These treatments did not cause tubule degeneration/necrosis; however, tubule regeneration was observed for hexachlorobenzene and lomustine, and several compounds caused mild tubule dilatation. Overall, our results show that cisplatin-induced gene expression perturbations in our novel 3D in vitro platform resemble and can predict those observed *in vivo* for rat kidney.

**Table 4:** Most similar rat kidney profiles to 2D and 3D RPTEC-TERT1 (monolayer vs. tubuloid) obtained from TXG-MAPr application.

query, 48 hr gene expression	TG kidney profile*	Pearson similarity	rank
cisplatin-3D	D:cisplatin-1d-3mg/kg	0.50	1
cisplatin-3D	cadmium-2d-3D_kidney_culture	0.48	2
cisplatin-3D	D:cisplatin-0.375d-3mg/kg	0.48	3
cisplatin-3D	D:cisplatin-0.25d-1mg/kg	0.38	4
cisplatin-3D	D:cisplatin-0.375d-1mg/kg	0.38	5
cisplatin-3D	D:cisplatin-4d-1mg/kg	0.37	6
cisplatin-3D	D:cisplatin-0.25d-3mg/kg	0.37	7
cisplatin-3D	D:puromycin aminonucleoside-0.25d-120mg/kg	0.36	8
cisplatin-3D	D:carboplatin-0.375d-100mg/kg	0.35	9
cisplatin-3D	D:cisplatin-0.125d-3mg/kg	0.34	10
cisplatin-2D	cisplatin-0.08d-3D_kidney_culture	1.00	1
cisplatin-2D	N:17-methyltestosterone-15d-100mg/kg	0.39	2
cisplatin-2D	N:cyclophosphamide-0.25d-2mg/kg	0.28	3
cisplatin-2D	cadmium-2d-2D_kidney_culture	0.25	4
cisplatin-2D	R:hexachlorobenzene-0.375d-1000mg/kg	0.25	5
cisplatin-2D	N:doxorubicin-4d-0.1mg/kg	0.24	6
cisplatin-2D	N:acetaminophen-0.25d-1000mg/kg	0.21	7
cisplatin-2D	R:allyl alcohol-0.25d-10mg/kg	0.19	8
cisplatin-2D	N:valproic acid-15d-450mg/kg	0.19	9
cisplatin-2D	N:imipramine-4d-30mg/kg	0.18	10
cadmium-3D	cisplatin-2d-3D_kidney_culture	0.48	1
cadmium-3D	N:17-methyltestosterone-15d-100mg/kg	0.42	2
cadmium-3D	N:bucetin-0.125d-2000mg/kg	0.41	3
cadmium-3D	N:monocrotaline-1d-30mg/kg	0.41	4
cadmium-3D	N:bromobenzene-0.125d-300mg/kg	0.41	5
cadmium-3D	N:ciprofloxacin-0.125d-300mg/kg	0.41	6
cadmium-3D	N:bucetin-0.125d-1000mg/kg	0.40	7
cadmium-3D	R:hexachlorobenzene-4d-100mg/kg	0.40	8
cadmium-3D	N:bromobenzene-0.375d-30mg/kg	0.39	9
cadmium-3D	R:lomustine-0.125d-6mg/kg	0.39	10
cadmium-2D	cisplatin-2d-2D_kidney_culture	0.40	1
cadmium-2D	cisplatin-0.08d-2D_kidney_culture	0.40	2
cadmium-2D	R:cyclophosphamide-0.125d-15mg/kg	0.31	3
cadmium-2D	N:bromoethylamine-0.25d-20mg/kg	0.27	4
cadmium-2D	D:cisplatin-0.125d-3mg/kg	0.27	5
cadmium-2D	N:acetaminophen-0.25d-1000mg/kg	0.26	6
cadmium-2D	cisplatin-0.08d-3D_kidney_culture	0.25	7
cadmium-2D	N:cyclophosphamide-0.25d-2mg/kg	0.25	8
cadmium-2D	N:17-methyltestosterone-15d-100mg/kg	0.25	9
cadmium-2D	D:thioacetamide-4d-45mg/kg	0.25	10

The gene expression profiles most similar to the query profiles appear in column 1; profiles containing “kidney_culture” are those we uploaded to the application; other profiles are rat kidney treatments from TG-GATES. The notation for TG-GATEs profiles is: Phenotype:Compound-duration of dosing in days-dose in mg/kg. Phenotypes were obtained by reviewing histology findings for repeat dose studies of 4–29 day duration where D denotes that the treatment causes tubule degeneration/necrosis; R denotes treatments that cause regeneration only, and N denotes treatments without these effects (but possibly other lesions); (from https://toxico.nibiohn.go.jp/open-tggates/english/pathology_search/screen7/pathology?finding_id=FI-K0400&organ_id=ORGA0020; accessed 7/14/2021).

### Image analysis of nephrotoxicity

**Fig 5:**
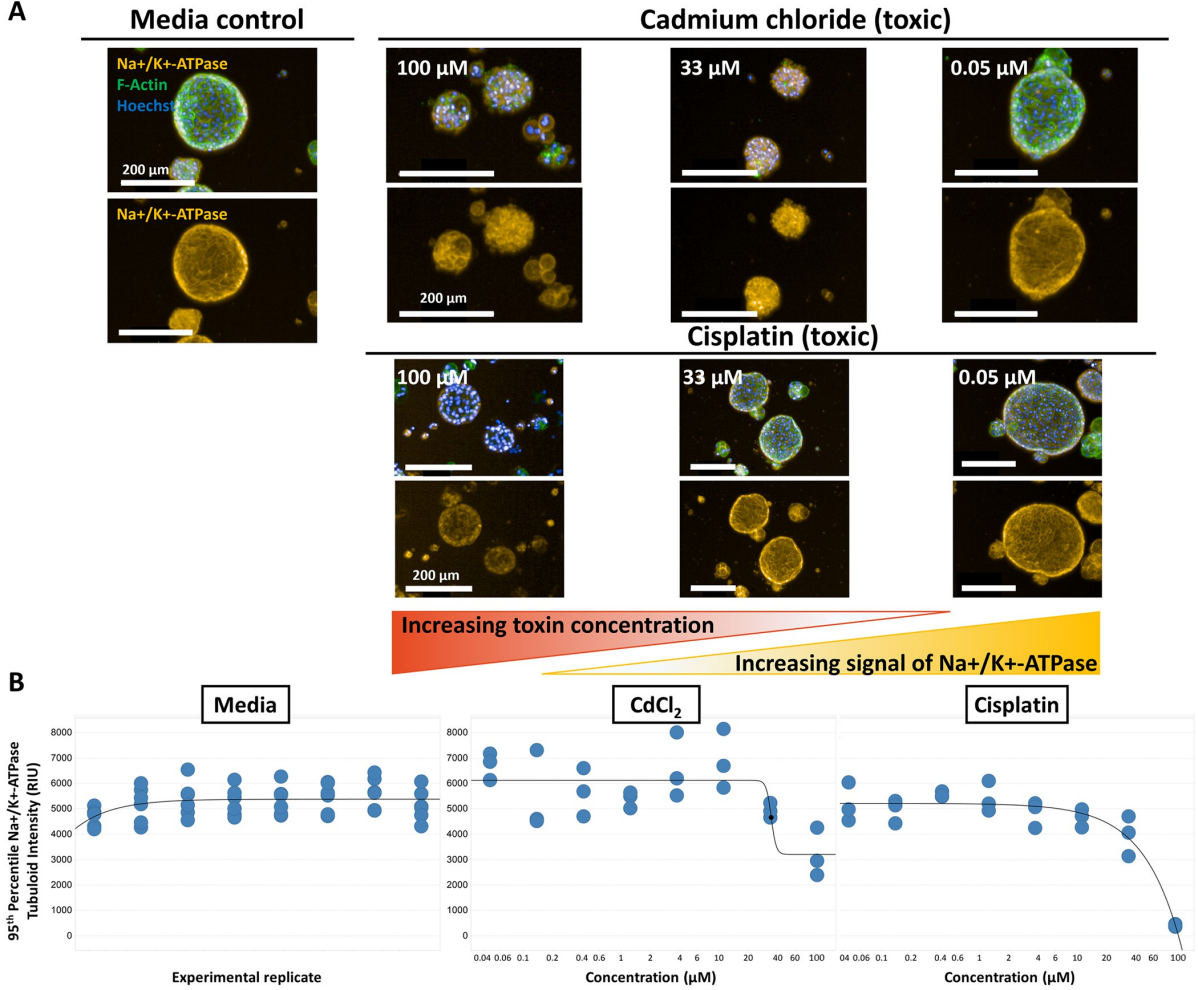
Nephrotoxicity image analysis of 3D tubuloids as a potential phenotypic screening tool. (A) Na+/K+-ATPase signal intensity in relative intensity units (RIU) diminishes with increasing dose of known nephrotoxins at 24-hour post-dose compared to media (non-toxic) control, scale bars are 200 μm. (B) Image quantification of the 95th percentile Na+/K+-ATPase Tubuloid intensity with respect to concentration at 24 hours post-dose.

Following incubation with nephrotoxins (cadmium chloride or cisplatin), there was a marked decrease in signal intensity in relative intensity units (RIU) of the transporter Na+/K+-ATPase. The tubuloid’s Na+/K+-ATPase signal intensity with respect to concentration was quantified (Fig 5). Further, the highest doses of compound may impact tubuloid morphology.

## Discussion

### 2D vs 3D cell-based assays for drug discovery

Drug discovery faces immense challenges due to unforeseen toxicity that cannot be underemphasized [3]. A key factor is the lack of predictive, *in vitro*, human cell-based assay. Given the growing understanding that renal assays in monolayer available to date lack relevance, shifting efforts to 3D systems is our strategy and has been gaining significant interest in the research community. However, in order to be relevant in drug discovery environment, these systems need to be not only clearly physiologically relevant and translatable to human physiology (i.e., 3D versus 2D, human versus mouse, etc.), they also need to be feasible for high-throughput assessments. While it is also generally agreed that cell-based assays do introduce more complexity to screening, nevertheless they will likely be required to acquire most predictive test systems [37].

The novel 3D tubuloid model we are introducing here demonstrated multiple advantages over 2D monolayer culture. The most key aspect of the 3D tubuloids, displayed in results, describe the greater similarity of 3D tubuloids to available benchmarks including human tissue and a *in vivo* rat database. This similarity can be attributed to the morphological route of how a potential drug would affect relevant cells. Since the tubuloid self-organizes into a complex structure with a hollow lumen, is clearly polarized with primary cilia facing the apical lumen, and integrin-beta 1 (among several other markers including Na+/K+-ATPase) presenting basolaterally, it can be assumed that compounds would be transported across the cell membrane (from basal to apical side) and into the lumen—a key feature of proximal tubules. The 2D monolayers will lack this aspect, with the compound directly interfacing with the apical side of the cell, without anticipated transport. This crucial difference likely results in the mismatch in sensitivity to compounds—we may be underestimating the nephrotoxic potential when we only screen in 2D. The lack of transporters in 2D systems has been described as a disadvantage of the system [21].

The 3D tubuloids also demonstrated more sensitivity than 2D with respect to secreted injury proteins—again, a key advantage to the 3D system. Further a reliable protein-based assays, with possible biomarker candidates justified in Table 5, could serve as a secondary assay in addition to image-based screening, which might be more feasible for high-throughput screens in drug discovery settings.

**Table 5:** Injury biomarker selection and rationale.

Biomarker	Location expressed	Justification
Kidney injury molecule 1 (KIM-1/Havcr1)	Proximal tubule	Upregulated following tubular injury [38]
Osteopontin (OPN)	Proximal tubule Distal tubule	Upregulated in both acute and chronic tubular injury [38]
Alpha-2-macroglobulin (A2M)	Plasma	Increased A2M levels seem to be correlated with nephrotic syndrome [39]
Interleukin-18 (IL-18)	Urine	Synthesized by proximal tubules, linked to predicting acute kidney injury *in vivo* [40,41]
Chemokine (C-X-C motif) ligand 1 (CXCL1)	Mature immune cells (neutrophils)	Inflammatory chemokines may be upregulated during kidney injury [42]

### Network analysis/RNASeq

Of note, cadmium chloride was not present in the rat kidney database (used for the comparisons in Table 3 via TG-GATES), therefore there was not an opportunity for cadmium toxicities to be matched with our *in vitro* data as cisplatin was. However, cadmium is a known nephrotoxin, and has been linked to tubule degeneration following long (>1 month) dosing regimens [43]. However, in general, the RNASeq/TG-GATES comparisons demonstrated higher incidences of matches to the 3D tubuloids (cisplatin, 48 hours) to *in vivo* rat kidney data that also were cisplatin-treated. The 2D samples (cisplatin, 48 hours) matched once with a cisplatin study, but the rest of the results were varied. In general, the 3D results associated often with “D” denotations, meaning that the treatment caused tubule degeneration/necrosis, whereas 2D results were most often associated with “N” denotation (no degeneration/necrosis or regeneration, but possibly other lesions).

Treatments of ≤1 day duration from TG-GATEs were often performed at higher doses than those for ≥ 4 day duration. For example, cisplatin 3mg/kg was not studied ≥4 days, and we infer the occurrence of tubule degeneration at 3 mg/kg based on results at the lower doses of 0.3 and 1 mg/kg, both of which resulted in tubule injury at multiple time points ranging from 4–29 days. Since the time-scale of the RNASeq experiment was relatively short (48 hours was the maximum duration of nephrotoxin incubation), it could be thought of as a study of “acute” injury—aligning with the incidences of matches with cisplatin injury in 3D, which is thought to cause acute injury, whereas cadmium injury may be more reflective of chronic injury [43–45].

### Nephrotoxicity screen

High-content imaging has gained significant interest in recent years in the field of drug discovery for phenotypic screening, converging upon an increased effort in human, primary, and/or 3D systems or co-cultures [46]. Na+/K+-ATPase has been linked to renal tubular injury, and its impairment contributes to a disruption of key renal functional pathways [47,48]. Previous study has identified changes on immunofluorescence of Na+/K+-ATPase in rat proximal tubule cells upon exposure to cadmium [49].

We hypothesized that upon induced injury with cadmium chloride or cisplatin, there could be a reduction in signal intensity observable by imaging. There was an observable difference of these compounds (cadmium chloride and cisplatin) intensity of Na+/K+-ATPase compared to the control. Two mechanisms that might be related to these differences might include a loss of cell polarity, or endocytosis [49]. Further study is needed to determine its robustness for high throughput assays, but this could be promising imaging approach for a 3D nephrotoxicity platform.

## Conclusions

To date, there have been few studies that have been adapted to high-throughput specifically to probe renal biology, although recent work has identified sophisticated and realistic screening strategies in 2D for later (repeat-dose) study in 3D [7,27]; as well as complementary work done on establishing iPSC-derived organoids for high-throughput [16]. Our work seeks to combine optimized screening approaches (*i*.*e*., a predictive compound set to reliably identify nephrotoxins) with 3D renal biology in a feasible manner through the use of a single human cell type, RPTEC-TERT1, formed into 3D tubuloids. The tubuloids described herein displayed a stronger degree of similarity to human kidney tissue and to *in vivo* toxicity experiments than RPTEC-TERT1 monolayers. In addition, this assay can be conducted reproducibly in 384-well plates, which enables high-throughput screening of possible novel therapeutic candidates. These cells are also amenable to gene editing, providing an opportunity to adapt the tubuloids for the study of genetic kidney disease, including polycystic kidney disease (PKD), where no reliable human 3D *in vitro* models exist. In conclusion, the high-throughput system we have produced demonstrates promise as a tool to identify nephrotoxicity in early stages of drug discovery with higher predictability than the current 2D paradigm. Furthermore, the tubuloids should be adaptable for renal drug discovery, and can be combined with engineered platforms (for example, microphysiological systems), to advance our understanding of human renal biology and identify potentially novel therapeutics or targets.

## Supporting information

S1 Fig RPTEC-TERT1 monolayer at D10 immunohistochemistry on various markers. RPTEC-TERT1 display several key biomarkers for proximal tubules including KIM-1, Aquaporin-1, Aquaporin-3, OCT2, OAT1, SGLT2, Na+/K+-ATPase, Integrin beta 1, Acetylated tubulin, Vimentin, Cytokeratin, Osteopontin and HMOX1. The RPTEC-TERT1 cells are also positive for laminin, which cannot be visualized well in the 3D tubuloids due to the encapsulation within laminin-rich matrigel (100 μm scales).
(TIF)

S2 Fig RPTEC-TERT1 monolayer culture at D10 stained for primary cilia. RPTEC-TERT1 cells demonstrate positive staining for primary cilia, visualized in 3-dimensions shows individual cilium pointed upwards (100 μm scales).
(TIF)

S1 Table Materials list. (DOCX)

S2 Table Antibody list. (DOCX)

S3 Table 15 marker Luminex panel and abbreviations. (DOCX)
