# Supplementary figures and images for: “3D, human renal proximal tubule (RPTEC-TERT1) organoids ‘tubuloids’ for translatable evaluation of nephrotoxins in high-throughput”
Source: PLoS One. 2022 Nov 21;17(11):e0277937. doi: 10.1371/journal.pone.0277937 (PMC9678317; doi:10.1371/journal.pone.0277937)

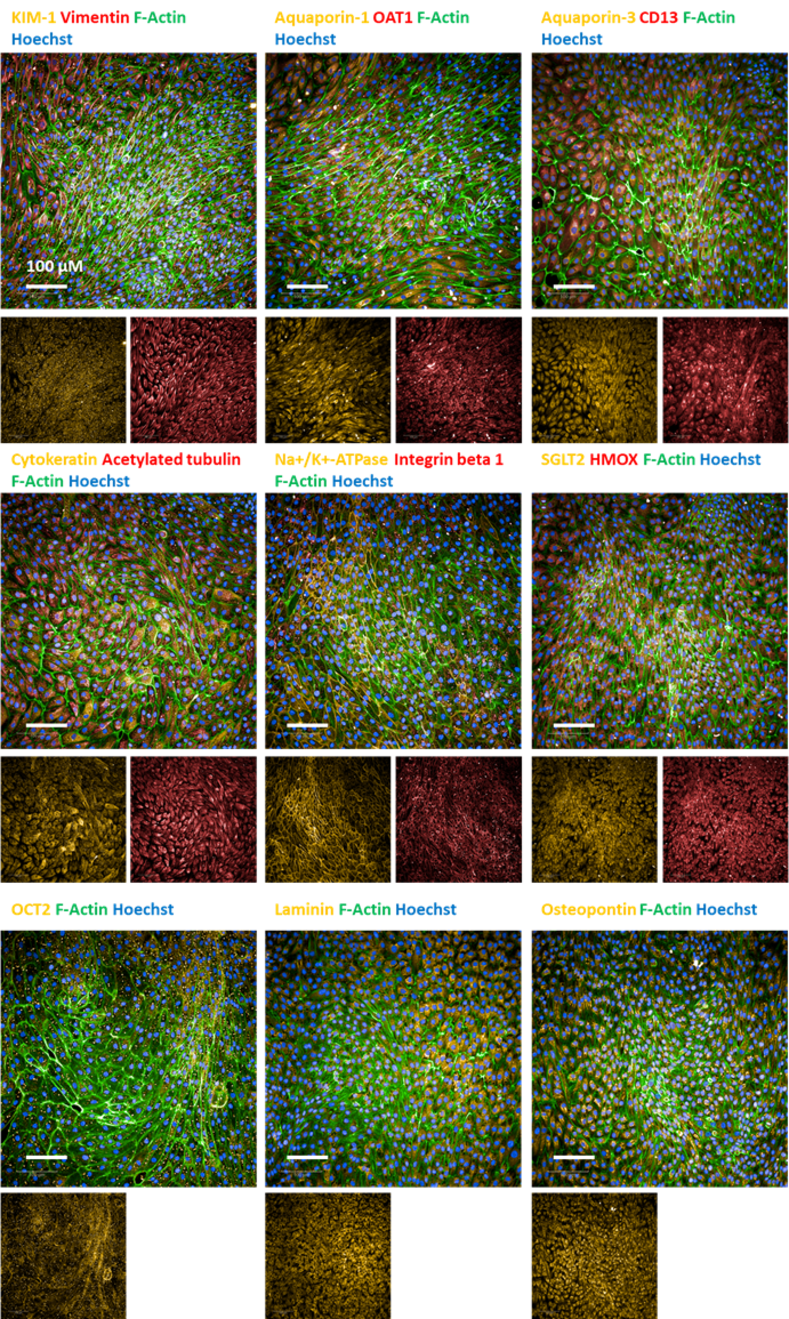

Supplement: S1 Fig — RPTEC-TERT1 display several key biomarkers for proximal tubules including KIM-1, Aquaporin-1, Aquaporin-3, OCT2, OAT1, SGLT2, Na+/K+-ATPase, Integrin beta 1, Acetylated tubulin, Vimentin, Cytokeratin, Osteopontin and HMOX1. The RPTEC-TERT1 cells are also positive for laminin, which cannot be visualized well in the 3D tubuloids due to the encapsulation within laminin-rich matrigel (100 μm scales). (TIF) [file pone.0277937.s001.tif]

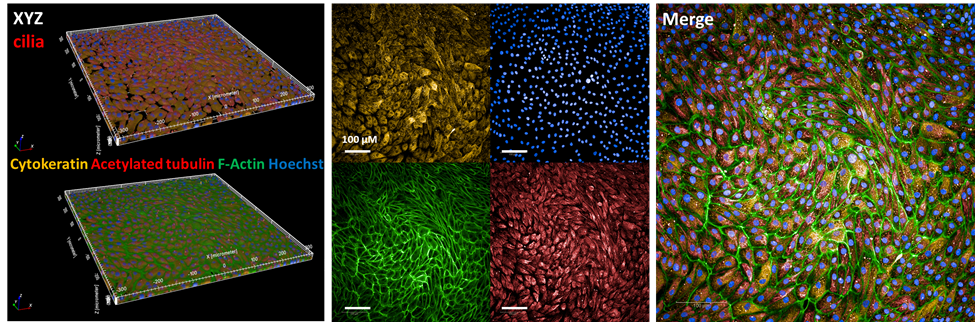

Supplement: S2 Fig — RPTEC-TERT1 cells demonstrate positive staining for primary cilia, visualized in 3-dimensions shows individual cilium pointed upwards (100 μm scales). (TIF) [file pone.0277937.s002.tif]
