# Supplementary material for: “3D, human renal proximal tubule (RPTEC-TERT1) organoids ‘tubuloids’ for translatable evaluation of nephrotoxins in high-throughput”
Source: PLoS One. 2022 Nov 21;17(11):e0277937. doi: 10.1371/journal.pone.0277937 (PMC9678317; doi:10.1371/journal.pone.0277937)
Supplement: S1 Table — (DOCX) [file pone.0277937.s003.docx]

**S1 Table. Materials list.**

| **Material** | **Vendor** | **Catalog #** |
| --- | --- | --- |
| Cell carrier plate | Perkin Elmer | 6057300 |
| Matrigel | Corning | 354230 |
| RPTEC/TERT1 cells | ATCC | CRL4031 |
| ATCC RPTEC/TERT1 Media | ATCC | 30-2006 |
| ATCC RPTEC/TERT1 Media growth kit | ATCC | 4007 |
| Accutase | Corning | 25-058-CI |
| G418 | VWR | 97064-358 |
| QIAShredder | Qiagen | 79656 |
| Cadmium chloride (CdCl2) | Rigaku Reagents | 1008153 |
| Trizol | ThermoFisher | 15596026 |
| LIVE/DEAD Viability/cytotoxicity reagent | ThermoFisher | L3224 |
| PrestoBlue viability reagent | ThemoFisher | A13261 |
| Cisplatin | Sigma | 479306 |
| Direct-zol RNA Miniprep | Zymo Research | R2050 |
| Pierce Rapid Gold BCA | ThermoFisher | A53227 |
