# Supplementary material for: “3D, human renal proximal tubule (RPTEC-TERT1) organoids ‘tubuloids’ for translatable evaluation of nephrotoxins in high-throughput”
Source: PLoS One. 2022 Nov 21;17(11):e0277937. doi: 10.1371/journal.pone.0277937 (PMC9678317; doi:10.1371/journal.pone.0277937)
Supplement: S2 Table — (DOCX) [file pone.0277937.s004.docx]

**S2 Table. Antibody list.**

| **Antibody/Dye** | **Type** | **Concentration** | **Product information** |
| --- | --- | --- | --- |
| Actubulin | Monoclonal | 1:500 | Millipore Sigma; Cat#MABT868 |
| Keratin | Polyclonal | 1:250 | Abcam (ab9377) |
| Phalloidin-488 | N/A | 1:1000 | Thermofisher (A12379) |
| Hoechst | N/A | 1:1000 | Thermofisher (H3570) |
| Anti-mouse-647 | Polyclonal | 1:500 | Thermofisher (A31571) |
| Anti-rabbit-555 | Polyclonal | 1:500 | Thermofisher (A32732) |
| Vimentin | Monoclonal | 1:500 | Abcam (ab8069) |
| TIM1/KIM1 | Polyclonal | 1:500 | Abcam (ab47635) |
| CD13 | Monoclonal | 1:100 | Abcam (ab7417) |
| AQP3 | Polyclonal | 1:100 | Abcam (ab125219) |
| AQP1 | Polyclonal | 1:100 | Abcam (ab15080) |
| Sodium Potassium ATPase | Monoclonal | 1:500 | Abcam (ab76020) |
| Integrin beta1 | Monoclonal | 1:250 | RnD Systems (MAB17781) |
| SGLT2 | Polyclonal | 1:100 | Abcam (ab37296) |
| HMOX1 | Monoclonal | 1:100 | Thermofisher (MA1-112) |
| OCT2 | Monoclonal | 1:100 | Abcam (ab170871) |
| OAT1 | Monoclonal | 1:100 | Thermofisher (Ma5-27677) |
| Osteopontin | Polyclonal | 1:100 | Thermofisher (22952-1-AP) |
