# Supplementary material for: “3D, human renal proximal tubule (RPTEC-TERT1) organoids ‘tubuloids’ for translatable evaluation of nephrotoxins in high-throughput”
Source: PLoS One. 2022 Nov 21;17(11):e0277937. doi: 10.1371/journal.pone.0277937 (PMC9678317; doi:10.1371/journal.pone.0277937)
Supplement: S3 Table — (DOCX) [file pone.0277937.s005.docx]

**S3 Table. 15 marker Luminex panel and abbreviations.**

| **15 marker panel (96 well)** | **Acronym (if applicable)** |
| --- | --- |
| Alpha 2-Macroglobulin | A2M |
| Adiponectin |  |
| Beta 2-Microglobulin | B2M |
| Calbindin D |  |
| Chemokine (C-X-C motif) ligand 1 | CXCL1 |
| Cystatin C |  |
| Lipocalin | NGAL |
| Osteopontin | OPN |
| Renin |  |
| Trefoil factor 3 | TFF3 |
| Kidney injury marker 1 | KIM-1/Havcr1 |
| Vascular endothelial growth factor | VEGF |
| Uromodulin |  |
| Interleukin 18 | IL-18 |
| Chemokine (C-C motif) ligand 2/Monocyte chemoattractant protein 1 | CCL2/MCP-1 |
